# Effects of later dinner timing on subsequent metabolic function and nocturnal sleep in healthy young women

**DOI:** 10.1186/s40101-026-00430-0

**Published:** 2026-04-13

**Authors:** Minori Enomoto, Shingo Kitamura, Taiki Kunieda, Taisuke Eto

**Affiliations:** 1 Department of Medical Technology, School of Health Sciences, Tokyo University of Technology, 5-23-22, Nishikamata, Ohta-Ku, Tokyo 144-8535 Japan; 2 Department of Sleep-Wake Disorders, National Institute of Mental Health, National Center of Neurology and Psychiatry, 4-1-1 Ogawa-Higashi, Kodaira, Tokyo 187-8553 Japan; 3 Department of Medical Technology and Clinical Engineering, Faculty of Health and Medical Sciences, Hokuriku University, 1-1 Taiyogaoka, Kanazawa, Ishikawa, 920-1180 Japan; 4 Department of Ophthalmology, Keio University School of Medicine, 35 Shinanomachi, Shinjuku-ku, Tokyo 160-8582 Japan

**Keywords:** Sleep, Meals, Food timing, Glucose, Continuous glucose monitoring

## Abstract

**Objectives:**

This study aimed to investigate the effects of dinner timing on subsequent sleep architecture and glucose metabolism in healthy young women, using objective and integrated physiological measures in a real-life setting.

**Methods:**

We conducted a randomized crossover trial with two dinner timing conditions: 1 h and 5 h before habitual bedtime. Each intervention lasted 6 days (Day 0 to Day 5), including a baseline day (Day 0) and 4 intervention days (Days 1–4). Dinner provided 709–740 kcal, with consistent macronutrient composition across conditions. Overnight sleep electroencephalography (EEG) was recorded on Day 0 and Day 4, and continuous glucose monitoring (CGM) was conducted throughout the experimental period. An oral glucose tolerance test (OGTT) was performed after waking on Day 5.

**Results:**

Thirteen healthy young women (21.4 ± 0.6 years) participated. On Day 4, the late-dinner condition (1 h before bedtime) resulted in significantly shorter total sleep time (TST, *p* = 0.013) and reduced sleep efficiency (SE, *p* = 0.040) and significantly higher wake after sleep onset (WASO, *p* = 0.017), Arousal Index (*p* = 0.041), number of stage-shifts (*p* = 0.016), and Stage-Shift Index (*p* = 0.003). The iAUC for postprandial glucose showed a significant interaction (*p* = 0.042), with lower values on Days 3 and 4 than on Day 1 (*p* = 0.090). OGTT results showed no significant changes.

**Conclusion:**

Dinner consumed 1 h before bedtime was associated with reduced sleep continuity and stability, while only transient changes in postprandial glucose dynamics were observed. In healthy young women, eating close to bedtime may affect sleep architecture, warranting further investigation.

**Supplementary Information:**

The online version contains supplementary material available at 10.1186/s40101-026-00430-0.

## Background

In recent years, increasing attention has been given to the impact of meal timing on human health [1, 2]. In particular, the later dinner timing has been identified as a risk factor for cancer, cardiometabolic risk, overweight/obesity, metabolic function, and body composition [3–5]. Additionally, several studies have reported that consuming dinner shortly before bedtime is associated with shorter sleep duration, reduced sleep quality, including increased sleep onset latency and decreased sleep efficiency [6, 7]. These findings have been reflected in the public health guidelines; for instance, the Ministry of Health, Labour and Welfare of Japan has incorporated this issue into its “Standard Health Check-up and Guidance Program,” which monitors habits such as eating dinner within 2 h of bedtime. However, the scientific basis for these recommendations has yet to be fully established, likely due to various potential confounding factors [8], and experimental evidence demonstrating the direct effects of a later dinner on metabolic function and sleep architecture remains insufficiently established.

Theoretically, late-evening eating is hypothesized to induce circadian misalignment and disrupt metabolic and sleep homeostasis. Eating during the biological night, when melatonin levels are elevated, may impair glucose tolerance via reduced insulin secretion and/or sensitivity [9]. Late meals may also alter postprandial thermogenesis [10] and exacerbate nocturnal gastroesophageal reflux that disrupts sleep [11], potentially increasing autonomic arousal and sleep fragmentation and thereby interacting with nocturnal glucose regulation.

However, despite these plausible mechanisms, empirical evidence concerning associations between dinner timing and sleep or metabolic parameters remains inconsistent. For instance, while some reports indicate that a late dinner impairs sleep quality, other longitudinal or experimental studies have found no significant association between eating shortly before bed and impaired sleep efficiency or longer sleep onset latency [12, 13]. Similarly, the associations between dinner timing and metabolic functions have been reported, but negative findings have also been shown [14]. Furthermore, many of these studies were limited by single-day interventions, reliance on self-report measures, and/or settings in strictly controlled laboratory environments. Consequently, the impact of evening meal timing on subsequent nocturnal sleep architecture and glucose dynamics has not been rigorously investigated using objective measurements such as electroencephalography (EEG) and continuous glucose monitoring (CGM) in naturalistic conditions. Therefore, to elucidate the relationship between meals, sleep, and metabolism, experimental validation incorporating objective, multifaceted assessments in real-life settings is essential.

Epidemiology and scoping reviews suggest that the sleep‑ and glycemia-related effects of evening meal timing can be sex-dependent, with signals often larger or more consistent in women [15–18]. It has also been reported that there are gender and physical differences in glucose tolerance [19]. Previous reports have suggested that young individuals with lower body weight tend to maintain more regular meal timing [20]. Based on this evidence, we recruited young women with relatively lean body types as participants in the present study. Furthermore, female-specific biology [21]—especially fluctuations in sex steroids across the reproductive years—modulates sleep architecture, thermoregulation, autonomic tone, and glucose handling, plausibly amplifying susceptibility to late-evening eating.

The purpose of this study was to examine the effects of dinner timing on subsequent sleep architecture and glucose metabolism in healthy young women, using objective and integrated physiological measures in real-life settings. We hypothesized that consuming dinner 1 h before bedtime, compared to 5 h before, would (1) impair objective sleep quality and (2) worsen postprandial glycemic control and insulin sensitivity. To test this hypothesis, we conducted a crossover trial in healthy young women with two dinner timing conditions: 1 h and 5 h before their habitual bedtime. The experiment was conducted in a home-based environment and included sleep EEG monitoring using a portable two-channel device, CGM throughout the study, and oral glucose tolerance test (OGTT) at the end of each intervention period. This study, by exploring the interplay of meals, sleep, and metabolism under conditions that closely mimic daily life, is expected to provide significant scientific and clinical insights for the prevention of lifestyle-related diseases and the promotion of sleep hygiene.

## Methods

### Participants

Fourteen female undergraduate students at the Tokyo University of Technology were recruited. The sample size was determined based on a previous study that investigated the effect of meal timing on total sleep time (TST). Inclusion criteria were as follows: (1) healthy female university students aged 20–25 years; (2) body mass index (BMI) between 18.5 and 24.9 kg/m^2^; (3) regular sleep–wake cycle (bedtime between 23:00 and 02:00, wake-up time between 06:00 and 09:00). Exclusion criteria were as follows: (1) history of any diagnosed sleep disorders, cardiovascular disease, or metabolic disorders (e.g., diabetes); (2) an Apnea–Hypopnea Index (AHI) of ≥ 5 events/hour on the screening polysomnography; (3) being a shift worker or traveling over time zones within 6 months before experiments; (4) current smoker; (5) habitual consumer of sleep-affecting medications. We assessed if the participants met the inclusion criteria (1)–(3) and did not meet the exclusion criteria (1) and (3)–(5) through a preliminary interview. The participants were screened for sleep disorders using overnight polysomnography for confirmation of exclusion criteria (1) and (2). The study was approved by the Tokyo University of Technology Institutional Review Board (IRB approval No. E23HS-001). All participants provided written informed consent prior to their participation in the study.

The experiments were conducted between May and June 2021 and between May and June 2023, with all participants completing the entire protocol within 1 month.

### Protocol

This intervention trial with two conditions adopted a crossover design, and all participants participated in both conditions. Each intervention lasted 6 days (Day 0 to Day 5), including a baseline day (Day 0) and four intervention days (Day 1–4), followed by an oral glucose tolerance test (OGTT) on Day 5. Height, weight, and blood pressure were measured before each condition. An automatic blood pressure monitor (Vital Note TM-2580, A&D Company, Tokyo, Japan) was used to measure blood pressure.

**Fig. 1:**
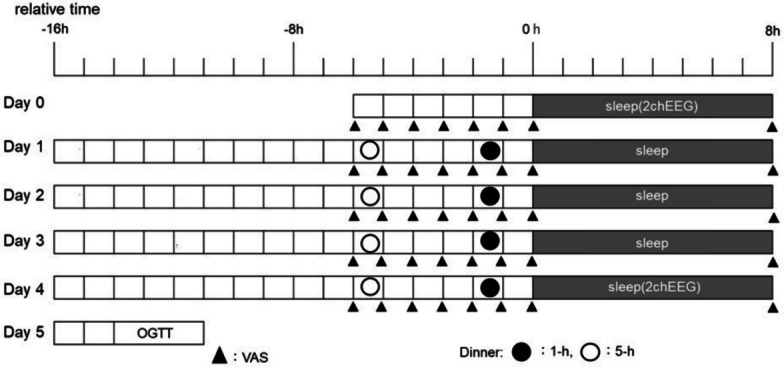
Experimental protocol. The intervention period included two dinner timing conditions: an early dinner condition (the 5-h condition: dinner consumed 5 h before lights out) and a late dinner condition (the 1-h condition: dinner consumed 1 h before lights out). The observation schedule was determined based on the sleep diary recorded during the week preceding the experiment and was expressed in relative time, with the average habitual bedtime set at 00:00

Participants were randomly assigned to one of the two condition sequences (5-h to 1-h or 1-h to 5-h) to ensure a counterbalanced order. Two periods were separated by a washout period of at least 1 week, with the total duration for each participant not exceeding 4 weeks. The experimental protocol is illustrated in Fig. 1.

The intervention period included two dinner timing conditions: an early dinner condition (the 5-h condition: dinner consumed 5 h before lights out) and a late dinner condition (the 1-h condition: dinner consumed 1 h before lights out). Data collection was conducted in participants’ homes to minimize disruption to daily life. During the washout interval between conditions, participants were instructed to avoid major deviations from their usual sleeping and eating patterns. The observation schedule was determined based on the sleep diary recorded during the week preceding the experiment and was expressed in relative time, with the average habitual bedtime set at 00:00. The participants also completed the Pittsburgh Sleep Quality Index [22], the Morningness–Eveningness Questionnaire [23], and the Epworth Sleepiness Scale [24] when recording their sleep diaries.

Napping and night shift work were prohibited throughout the study. Participants were instructed to refrain from strenuous exercise after dinner. Participants were also instructed to refrain from snacking, alcohol consumption, smoking, and caffeine intake.

### Dietary control

Dinner on Day 0 (pre-intervention) was consumed ad libitum in terms of both timing and content. On Days 1–4 (intervention days), dinner content was standardized across participants. In the 5-h condition, participants were instructed to consume dinner over a 30-min period beginning 5.5 h and ending 5 h before bedtime. In the 1-h condition, dinner was consumed in a similar manner between 1.5 and 1 h before bedtime.

The dinner menus, which were provided by commercially available food delivery companies, consisted of a pre-packaged main dish, white rice, a protein bar, and vegetable juice. The meals contained approximately 700 kcal (ranged 709–740 kcal), corresponding to one-third of the daily energy intake for an average adult Japanese woman, and were balanced in macronutrient composition (protein 15%, fat 20%, and carbohydrate 65% per total calories). The types of main dishes were identical throughout each intervention period.

Breakfast and lunch were limited to consume with the window of 16–14 h and 12–10 h prior to bedtime, respectively, while the content of breakfast and lunch was self-selected. Participants recorded the timing and dietary intake of each of their breakfast and lunch immediately after their meal to facilitate dietary monitoring.

## Measurements

### Sleep EEG

Sleep EEG recordings were conducted using a validated [25] portable two-channel EEG device (ZA-X, Proassist, Ltd., Osaka, Japan) on the night of Day 0 (pre-intervention) and Day 4 (the last day of the intervention days). Recordings were scheduled according to each participant’s habitual sleep duration, with the habitual bedtime (hBT) used as the reference start time and total recording time (TRT) ranging from 6 to 9 h. Participants self-administered the EEG device, including electrode placement and initiation/termination of the recording. They were also instructed to document their actual bedtime, final awakening time, and any time spent out of bed. Sleep stages (Stage N1, N2, N3, R, and W) were scored in 30-s epochs based on the criteria of the American Academy of Sleep Medicine Manual for the Scoring of Sleep and Associated Events [26].

### CGM

Participants were instructed to wear a CGM sensor (FreeStyle Libre Pro, Abbott Japan LLC, Tokyo, Japan) throughout the intervention period. The CGM sensor, which weighs 5 g, was placed on the back of the participant’s nondominant upper arm. It stores real-time glucose measurements between 40 and 500 mg/dL at 15-min intervals. FreeStyle Libre Pro was approved by the FDA and validated against blood glucose, and the mean absolute relative difference (MARD) ranged from 11.4 to 14.8, with ~90% values falling into the consensus error grid Zone A or Zone B [27, 28]. Immediately after sensor placement, interstitial glucose readings were confirmed to ensure proper attachment prior to data collection. Participants were advised not to remove the sensor until the end of the intervention period.

### OGTT

OGTT, a standard clinical method for assessing glucose intolerance and diagnosing diabetes mellitus [29], was conducted on Day 5. After completing the final intervention dinner, participants fasted for at least 10 h and then visited the laboratory at Tokyo University of Technology to undergo the 3-h OGTT. Participants ingested 75 g of glucose (Trelan G, Yoshindo Inc. Toyama, Japan) and were followed up to 3 h in semi-recumbent posture. The CGM monitoring continued throughout the OGTT [30], and venous blood samples were collected at baseline (fasting) and 30 min after glucose ingestion as well. Plasma glucose and serum insulin concentrations at baseline and 30 min after glucose ingestion were analyzed by Hoken Kagaku, Inc (Kanagawa, Japan).

### Subjective sleepiness and hunger

Subjective assessments were conducted using Visual Analogue Scale (VAS) from Day 0 to Day 4. Participants completed VAS ratings at hourly intervals, beginning 6 h before bedtime and continuing until bedtime (7 time points in total). Two subjective parameters were evaluated: sleepiness (“very sleepy” to “not sleepy at all”) and hunger (“most hungry ever” to “not hungry at all”).

Additionally, a VAS evaluation was conducted once each morning immediately after awakening (Days 1–5).

## Data analysis

### Sleep parameters

The following sleep parameters were derived from the scored EEG data: total recording time (TRT), total sleep time (TST), sleep efficiency (SE = TST/TRT × 100), sleep latency (SL), REM latency, percent of TST spent in each stage (time in each stage (N1, N2, N3, and R)/TST × 100), wake after sleep onset (WASO), the Arousal Index (Arousals/h), Stage-Shift, Stage-Shift Index (Stage-Shift/h). After scoring the sleep stage each night, we counted the total number of changes in sleep stage between falling asleep and final awakening (Stage-Shift) [31]. A Stage-Shift Index (/h) was obtained by normalizing the total number of Stage-Shift to sleep period time (SPT).

For each variable, values obtained on Days 0 and 4 under the 1-h and 5-h conditions were analyzed using a linear mixed-effects model, with Day 4 as the dependent variable, CONDITION (1-h vs. 5-h) as a fixed effect, baseline value (Day 0) as a covariate, and participant ID as a random intercept. Pairwise comparisons between 1-h and 5-h adjusted for Day 0 were performed using estimated marginal means. Normality was assessed with the Shapiro–Wilk test. Parameters that violated normality assumptions were Box-Cox transformed [32] before analysis.

### Sleepiness and hunger

Subjective sleepiness and hunger were analyzed with a three-way mixed-effects ANOVA (CONDITION, DAY, and TIME as fixed factors and ID as a random factor).

Ratings for sleepiness and hunger upon awakening were analyzed with a two-way mixed-effects ANOVA (CONDITION and DAY as fixed factors and ID as a random factor).

### Frequency analysis

Frequency analyses were conducted using the *SleepSign* software (KISSEI COMTEC CO., LTD., Nagano, Japan). For frequency-domain analysis, Fast Fourier Transform (FFT) was conducted for the EEG channel. FFT was performed every 5 s using a Hanning window, and power values were calculated at 0.5 Hz intervals and δ band power (0.5–4 Hz) was calculated.

Outliers were identified and removed using the anomalize package in R [33]. The remaining δ power values were then normalized by z-score transformation and averaged for each 30-s epoch. NREM sleep cycles were identified according to the criteria proposed by Feinberg et al. [34], and mean δ power was calculated for each NREM cycle.

Mean z-scored δ power values were analyzed using a three-way mixed-effects ANOVA with CONDITION, DAY, and CYCLE as fixed factors and ID as a random factor.

### Postprandial glycemic response

Postprandial glycemic response was assessed using the incremental area under the curve (iAUC) for CGM glucose levels from 0 to 180 min after dinner on Days 1–4 under the 1-h and 5-h conditions. iAUC was calculated using the trapezoidal method. Data were analyzed using a mixed-effects model with CONDITION and DAY as fixed effects and ID as a random factor. When a significant interaction was observed, Holm’s multiple comparison test was conducted as a post hoc analysis.

#### OGTT

Blood glucose and insulin concentrations measured during the OGTT were used to compute the following indices of glucose metabolism:

$$\mathrm{HOMA} - \mathrm{IR} = \text{fasting insulin} (\upmu \mathrm{U}/\mathrm{mL}) \times \text{fasting glucose} (\mathrm{mg}/\mathrm{dL}) / 405.$$

$$\mathrm{HOMA} - \upbeta =\text{fasting insulin} (\upmu \mathrm{U}/\mathrm{mL}) \times 360/ [(\text{fasting glucose} (\mathrm{mg}/\mathrm{dL}) - 63].$$

$$\text{Insulinogenic Index} (\mathrm{IGI}) = [\text{insulin at 30 min}\ -\ \text{fasting insulin}] (\upmu \mathrm{U}/\mathrm{mL})/[\text{glucose at 30 min}\ -\ \text{fasting glucose}] (\mathrm{mg}/\mathrm{dL}).$$

In addition, the iAUC for glucose from baseline to 180 min post-glucose load was calculated by the CGM glucose levels. All indices were compared between the 1-h and 5-h conditions using one-way mixed-effects ANOVA with CONDITION as the fixed effect and ID as a random factor.

## Statistics

All results are expressed as mean ± standard deviation (SD). Statistical analyses were performed using R version 4.3.1 (R Core Team) with the following R packages: anomalize 0.3.0 [35], nls2 v.0.3.3 [36]. A *p*-value of < 0.05 was considered statistically significant.

## Results

### Demographic parameters

Fourteen participants were initially recruited and completed both trial conditions. Of these, one subject was excluded due to a sleep onset latency exceeding 60 min. As a result, data from 13 participants were included in the final analysis. Demographic information for these 13 participants is presented in Table 1.

**Table 1:** Demographic data

Sex(M/F)	0/13
Age (y)	21.41 ± 0.60
Weight (kg)	50.88 ± 6.39
Height (cm)	156.16 ± 5.47
BMI	21.00 ± 2.37
Systolic blood pressure (mmHg)	103.57 ± 13.21
Diastolic blood pressure (mmHg)	65.21 ± 8.95
Habitual bedtime	0:30 ± 0:51
Habitual dinner time	19:28 ± 0:46
PSQI score	4.08 ± 2.62
MEQ score	48.62 ± 10.77
ESS score	9.77 ± 3.31

*PSQI* Pittsburgh Sleep Quality Index, *MEQ* Morningness-Eveningness Questionnaire, *ESS* Epworth sleepiness scale

### Sleep parameters

Sleep parameters for each condition and day are summarized in Table 2. Except for %Stage R (as a percentage of TST), all other variables were Box–Cox transformed prior to statistical analysis. The values reported in Table 2 represent the raw (pre-transformation) data for interpretability.

**Table 2:** Sleep parameters per condition and day

	1-h		5-h		CONDITION	
	Day 0	Day 4	Day 0	Day 4	*t*	*p*
Lights out	0:28 ± 0:53	0:28 ± 0:52	0:33 ± 0:49	0:19 ± 0:49	−1.06	0.311
Lights on	8:17 ± 1:03	8:15 ± 0:51	8:11 ± 1:28	8:13 ± 0:52	−0.62	0.548
TRT (total recording time), min	469.38 ± 17.33	467.23 ± 21.96	469.14 ± 27.00	474.83 ± 15.54	−0.44	0.667
SL (sleep latency), min	17.58 ± 20.27	19.43 ± 22.07	21.92 ± 20.65	15.04 ± 8.40	0.59	0.566
TST (total sleep time), min	437.23 ± 22.22	415.15 ± 35.83	429.50 ± 24.11	441.21 ± 22.43	−2.97	0.013
REM latency, min	77.08 ± 16.31	95.23 ± 25.16	95.04 ± 41.32	90.17 ± 21.36	−0.92	0.382
%Stage N1 (/TST)	4.51 ± 2.83	5.95 ± 3.65	5.02 ± 3.08	4.72 ± 3.40	1.65	0.128
%Stage N2 (/TST)	37.73 ± 11.80	38.61 ± 8.29	39.42 ± 5.42	42.67 ± 6.11	−2.40	0.040
%Stage N3 (/TST)	30.46 ± 10.13	32.04 ± 11.04	31.93 ± 8.59	28.04 ± 8.51	1.33	0.212
%Stage R (/TST)	25.46 ± 2.97	23.16 ± 4.32	23.63 ± 4.19	24.57 ± 4.07	−0.92	0.382
SE (sleep efficiency), %	93.17 ± 3.68	91.47 ± 4.90	88.63 ± 7.40	92.94 ± 4.21	−2.17	0.048
WASO (wake after sleep onset), min	15.15 ± 8.73	29.00 ± 16.84	14.14 ± 9.25	15.58 ± 10.80	2.84	0.017
Arousal Index (/h)	5.33 ± 2.45	6.61 ± 2.14	5.36 ± 1.94	5.37 ± 1.78	2.31	0.041
Number of stage-shift	89.46 ± 17.54	109.23 ± 16.92	93.36 ± 26.44	92.58 ± 24.81	2.88	0.016
Stage-Shift Index (/h)	11.89 ± 2.44	14.76 ± 2.24	12.60 ± 3.53	12.11 ± 3.15	3.99	0.003

Values are before Box-cox transformation and represent mean ± sd. All statistics are calculated by Box-cox transformation except for %Stage R(/TST). For each variable, values obtained on Days 0 and 4 under the 1–h and 5–h conditions were analyzed with a linear mixed-effects model fitted with day 4 as the dependent variable, CONDITION (1 h vs. 5 h) as a fixed effect, baseline value (Day 0) as a covariate

A linear mixed-effects model revealed significant interaction effects for several key sleep parameters (Table 2). Significant interaction effects were found for the following sleep parameters: TST: estimate = −32.97, SE = 11.08, t(10) = −2.97, *p* = 0.013, WASO: estimate = 2.12, SE = 0.74, t(10) = 2.84, *p* = 0.017, SE: estimate = −6.78 × 10^17^, SE = 3.12 × 10^17^, t(13) = −2.17, *p* = 0.048, Arousal Index(/h): estimate = 0.13, SE = 0.06, t(11) = 2.31, *p* = 0.041, number of Stage-Shift: estimate = 664.03, SE = 223.68, t(10) = 2.88, *p* = 0.016, Stage-Shift Index(/h): estimate = 25.40, SE = 6.36, t(10) = 3.99, *p* = 0.003, percent of TST in Stage N2: estimate = −2521.99, SE = 1050.92, t(9) = −2.40, *p* = 0.040.

No significant main or interaction effect was observed for Lights Out (estimate = −45,027.84, SE = 42,444.69, t(11) = −1.06, *p* = 0.311), Light On (estimate = −0.49, SE = 0.78, t(10) = −0.62, *p* = 0.548), TRT (estimate = −4.07 × 10^21^, SE = 9.20 × 10^21^, t(11) = −0.44, *p* = 0.667), REM latency(estimate = −1.14, SE = 1.25, t(10) = −0.92, *p* = 0.382), and percent of TST spent in each stage except Stage N2(%Stage N1; estimate = 0.57, SE = 0.34, t(11) = 0.65, *p* = 0.128,%Stage N3; estimate = 0.02, SE = 0.01, t(11) = 1.33, *p* = 0.212, %Stage R; estimate = −1.14, SE = 1.25, t(10) = −0.92, *p* = 0.382). No differences were observed between the predefined conditions (Lights out, Lights on, and TRT) in schedule-related variables, indicating good adherence to the experimental protocol.

### Sleepiness and hunger

Subjective sleepiness increased progressively toward bedtime in both 1-h and 5-h, with no significant differences observed between conditions, days, or time points (*F* (6, 324) = 1.15, *p* = 0.335). For subjective hunger, ratings increased up to mealtime and decreased following food intake. A three-way mixed-effects ANOVA revealed a significant interaction effect (*F* (6, 324) = 8.91, *p* < 0.0001), although no main effect or interaction regarding condition was found.

Upon awakening, subjective sleepiness or hunger did not differ significantly across conditions (*F* (1, 33) = 1.09, *p* = 0.304 and *F* (1, 34) = 0.75, *p* = 0.394, respectively).Temporal changes in subjective sleepiness and hunger are shown in Supplementary Fig. S1.

### Frequency analysis

**Fig. 2:**
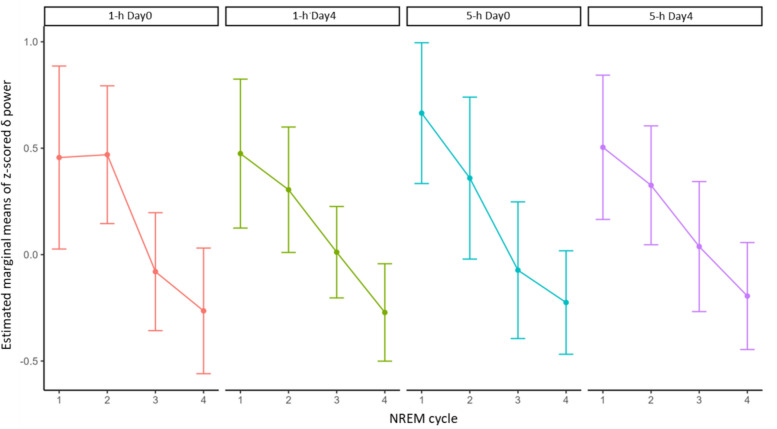
Changes in δ band (0.5–4 Hz) EEG power during NREM cycle. Data represented as estimated marginal means of z-scored power with 95% confidence interval

Z-scored mean δ power values across NREM cycles were compared using a three-way mixed-effects ANOVA (CONDITION, DAY, CYCLE). No significant three-way interaction was found (*F* (1, 172) = 0.83, *p* = 0.36). There were also no significant main effects of CONDITION (*F* (1, 172) = 1.03, *p* = 0.31) or DAY (*F* (1, 173) = 2.62, *p* = 0.11). However, a significant main effect of CYCLE was observed (*F* (1, 172) = 248.35, *p* < 0.001), indicating expected physiological variation in δ power across NREM cycles. Z-scored mean δ power values across NREM cycles were in Fig. 2.

### Postprandial glycemic response

The iAUC values from Days 1 to 4 for each dinner timing condition are presented in Table 3. A significant interaction effect was found (*F* (3, 84) = 2.85, *p* = 0.042), and post hoc comparisons indicated that iAUC on Days 3 and 4 tended to be lower than on Day 1 under 1-h (*p* = 0.090 for both comparisons).

**Table 3:** Variation in iAUC after dinner over 4 days

		1-h	5-h	CONDITION × DAY		
				*F*-value	*p*	Post hoc
iAUC (mg/dL*min) after dinner	Day 1	7050.00 ± 1619.51	5499.23 ± 2723.08	2.85	0.046	1-h Day 1 > 1-h Day 3, *p* = 0.090, 1-h Day 1 > 1-h Day 4, *p* = 0.090
	Day 2	6605.77 ± 1754.07	6006.92 ± 2524.40			
	Day 3	5102.31 ± 1348.26	6310.38 ± 2586.74			
	Day 4	5101.15 ± 1477.88	5158.85 ± 3014.37			

Postprandial glycemic response was assessed using the incremental area under the curve (iAUC) for glucose levels from 0 to 180 min after dinner on Days 1–4 under the 1 h and 5 h conditions. Data were analyzed using a mixed-effects model with CONDITION and DAY as fixed effects. Holm’s multiple comparison test was conducted as a post hoc analysis

#### OGTT

Results for HOMA-IR, HOMA-β, IGI, and iAUC under 1-h and 5-h are summarized in Table 4. A trend toward lower HOMA-IR under 5-h compared to 1-h was found (*F* (1, 11) = 3.70, *p* = 0.080) but did not reach statistical significance. No significant differences were found between conditions for HOMA-β, IGI, or iAUC.

**Table 4:** Blood glucose metabolism variables during OGTT

	1 h	5 h	CONDITION	
			*F*-value	*p*
HOMA-IR	1.77 ± 0.61	1.48 ± 0.72	3.70	0.080
HOMA-β(%)	118.81 ± 40.41	111.08 ± 48.35	0.35	0.563
IGI	1.22 ± 0.65	1.28 ± 0.71	0.22	0.648
iAUC(mg/dL*min)	6844.5 ± 1746.23	7879.5 ± 3203.47	1.42	0.263

The OGTT (oral glucose tolerance test) was performed on Day 5 and the final day. HOMA-IR = fasting insulin (µU/mL) × fasting glucose (mg/dL)/405, HOMA-β = fasting insulin (µU/mL) × 360/[fasting glucose (mg/dL) – 63], IGI (Insulinogenic Index) = [insulin at 30 min – fasting insulin] (µU/mL)/[glucose at 30 min – fasting glucose] (mg/dL). OGTT indices were compared between the 1-h and 5-h conditions using one-way mixed-effects ANOVA with CONDITION as the fixed effect

## Discussion

In this study, we investigated the effects of dinner timing on sleep using objective measures in healthy young adult women. The experimental protocol was customized for each participant by setting their usual average bedtime as relative time 0:00. On Day 4, the late-dinner condition (1 h before bedtime) resulted in significantly shorter TST, reduced SE and %Stage N2(/TST) and significantly higher WASO, Arousal Index, number of Stage-Shifts, and Stage-Shift Index.

Our results showed no significant variation in the distribution of sleep stages other than %Stage N2. Instead, eating dinner 1 h before bedtime was associated with increased WASO and reduced TST and SE, which are inversely correlated variables. These results suggest that rather than an increase in sleep duration, heightened nocturnal arousal—combined with an increase in the Arousal Index—led to a reduction in total sleep time. Increased arousal indicates reduced sleep continuity. Furthermore, the concurrent increase in the Stage-Shift Index suggests shorter stage durations and diminished sleep stability. Although no standardized criteria exist for sleep continuity or stability, these changes are consistent with sleep fragmentation. Previous studies have similarly reported increased sleep fragmentation, measured by combining the Arousal Index and Stage-Shift Index, in sleep disorders such as insomnia and restless legs syndrome [37]. In both insomnia and subjectively poor-sleep groups, wake frequency is often used as an indicator of sleep persistence, and stage-transition frequency as an indicator of sleep instability. These studies consistently reported reduced persistence (increased wake frequency) and reduced stability (increased stage transitions) [38]. Beyond sleep disorders, sleep fragmentation has been linked to adverse mental and physical consequences, including obesity, dysbiosis, atherosclerosis, and attention-deficit/hyperactivity disorder [39].

One plausible explanation for the increased arousal observed in our study is that delayed dinner timing caused postprandial hyperglycemia to extend into the sleep period, followed by a glucose decline during sleep. A previous study evaluating blood glucose fluctuations during overnight polysomnography in children with diabetes reported that rapid declines in blood glucose levels may trigger arousal responses [40]. In addition, experimental studies have shown increased arousal in healthy individuals when blood glucose levels are artificially lowered during sleep through insulin administration [41]. Consistent with these findings, the present results suggest an association between nocturnal fluctuations in blood glucose levels and micro-arousals.

Discrepancies regarding the impact of dinner timing in prior literature may arise from methodological differences, particularly in the target population and variations in assessment methods. In a cross-sectional study of athletes, nocturnal sleep duration was longer; however, increased wakefulness during the first half of the sleep period was observed [42]. Similarly, previous reports based on subjective assessments, such as the PSQI [42, 43], also support the objectively observed reduction in sleep quality reported in the present study. The robust effects observed in our study might be due to the rigorous setting of the dinner timing to 1 h before bedtime, which is a stricter condition than in studies allowing a 2–3 h interval or relying on self-reports.

Importantly, sleep onset latency did not differ between conditions, indicating that pre-sleep sleepiness was unaffected. Subjective ratings also showed a progressive increase in sleepiness toward bedtime under both conditions, with no between-condition differences. These findings suggest that dinner timing primarily influenced arousal after sleep onset. Consistent with our results, Lehmann et al. [44] reported no significant differences in sleep onset latency across dinner timing conditions.

In our study, participants in the late-dinner condition showed a tendency toward higher postprandial glucose levels on Day 1. Previous research has demonstrated that even a single day of late-dinner consumption can increase postprandial glucose AUC [45]. Postprandial glucose metabolism is regulated by circadian rhythms, and delaying meals can phase-delay these rhythms [46]. Moreover, circadian misalignment is associated with impaired glucose tolerance [47]. In our participants, the average interval between usual dinner time and bedtime was 4.34 ± 0.78 h. Thus, the 1-h dinner condition represented a substantial shift from habitual timing. Accordingly, the higher iAUC on Day 1 and its attenuation by Days 3–4 may reflect an acute perturbation after an abrupt shift in dinner timing with possible short-term adaptation over repeated exposure. However, because circadian phase and related mechanistic markers were not assessed, the underlying mechanisms remain speculative. The absence of such changes in the 5-h condition may be explained by its smaller deviation from habitual timing.

On Day 5, after the 4-day intervention, OGTT results showed no significant difference in HOMA-β or IGI, both indicators of insulin secretion. However, HOMA-IR, an index of insulin resistance, tended to be higher in the late-dinner group. In healthy Japanese adults, a HOMA-IR cutoff of 1.7 is considered indicative of lifestyle-related disease risk [48]. These results raise the possibility that consuming dinner immediately before bedtime could be associated with an increased risk of lifestyle-related diseases.

The intervention period in this study was 4 days. Previous reports with longer interventions have shown more pronounced effects. For example, an 8-week delayed-dinner protocol increased sleep latency by 25 min compared with a control schedule [49]. In contrast, Duan et al. found no significant differences in sleep outcomes when dinner timing was shifted for only 1 day [13]. Similarly, a study of gastroesophageal reflux disease patients found no acute effects of dinner timing on sleep [50]. These findings suggest that the effects of dinner timing may accumulate over repeated exposures, underscoring the importance of longitudinal studies. With regard to glucose tolerance, a single day of meal timing manipulation had no effect [51], whereas longer-term interventions demonstrated measurable impacts [49]. Taken together, our findings suggest that although glucose rhythms adapt to altered meal timing, repeated exposure to elevated postprandial glucose levels at sleep onset may impair sleep maintenance, with cumulative effects over time.

This study detected subtle alterations in sleep associated with eating dinner immediately before bedtime in healthy young women. However, several limitations should be noted. First, the study included only young women with small numbers, which may limit the generalizability of the findings. Second, although changes in REM sleep related to the luteal phase increase in body temperature [52, 53] and declines in subjective sleep quality around menstruation [54] have been reported, we prioritized collecting data under natural living conditions and did not control for participants’ menstrual cycles. Third, the intervention period was limited to 4 days. While this enabled us to evaluate effects across multiple days rather than a single intervention, further research is needed to clarify the impact of delayed meal timing when maintained over longer durations. Finally, irregular meal timing and meal skipping have been associated with poorer subjective sleep quality [55, 56] but we do not consider these effects. In this study, breakfast and lunch timing were standardized to 16–14 h and 12–10 h before bedtime, respectively; however, the content of meals other than dinner was not controlled.

## Conclusion

This comparative study employed an experimental protocol designed to reflect participants’ habitual bedtime, with dinner scheduled either 1 h or 5 h before bedtime for 4 consecutive days, to examine effects on sleep architecture and glucose metabolism. The results showed that, compared with the 5-h condition, consuming dinner 1 h before bedtime resulted in reduced SE, TST, increased WASO, and a higher Arousal Index. Combined with the observed increase in the number of Stage-Shifts, these findings suggest a tendency toward reduced sleep continuity and stability under the 1-h condition. In addition, consuming dinner 1 h before bedtime was associated with transient alterations in postprandial glucose dynamics, while no clear effect on overall glucose tolerance was observed. Taken together, within the context of this short-term intervention in healthy young women, these findings suggest that eating very close to bedtime may adversely affect certain aspects of sleep architecture and nocturnal glucose regulation. Further studies in broader populations and over longer durations are warranted to clarify the generalizability and clinical relevance of these observations.

## Supplementary Information

Additional file 1: Fig. S1. Subjective sleepiness and hunger. Sleepiness in the 1-h condition (a), sleepiness in the 5-h condition (b), hunger in the 1-h condition (c), and hunger in the 5-h condition (d). The blue line represents Day 0, and the orange line represents Day 4. According to the results of a three-way mixed-effects ANOVA, no significant interaction was observed for sleepiness, whereas a significant interaction was observed for hunger. Significance markers were added at points where multiple comparisons revealed significant differences between Day 0 and Day 4. Data are presented as mean ± SD. *p < 0.01, **p < 0.001.

## Data Availability

All data generated or analyzed during this study are included in this published article.

## Abbreviations

Arousals/h: Arousal Index
CGM: Continuous glucose monitoring
EEG: Electroencephalography
iAUC: Incremental area under the curve
IGI: Insulinogenic Index
OGTT: Oral Glucose Tolerance Test
SE: Sleep efficiency
Stage-Shift/h: Stage-Shift Index
SL: Sleep latency
SPT: Sleep period time
TRT: Total recording time
TST: Total sleep time
VAS: Visual Analogue Scale
WASO: Wake after sleep onset
