# Supplementary material for: Effects of later dinner timing on subsequent metabolic function and nocturnal sleep in healthy young women
Source: J Physiol Anthropol. 2026 Apr 13;45:12. doi: 10.1186/s40101-026-00430-0 (PMC13196191; doi:10.1186/s40101-026-00430-0)
Supplement: Supplementary file 1 — Additional file 1: Fig. S1. Subjective sleepiness and hunger. Sleepiness in the 1-h condition (a), sleepiness in the 5-h condition (b), hunger in the 1-h condition (c), and hunger in the 5-h condition (d). The blue line represents Day 0, and the orange line represents Day 4. According to the results of a three-way mixed-effects ANOVA, no significant interaction was observed for sleepiness, whereas a significant interaction was observed for hunger. Significance markers were added at points where multiple comparisons revealed significant differences between Day 0 and Day 4. Data are presented as mean ± SD. *p < 0.01, **p < 0.001. [file 40101_2026_430_MOESM1_ESM.docx]

**Fig. S1**

**
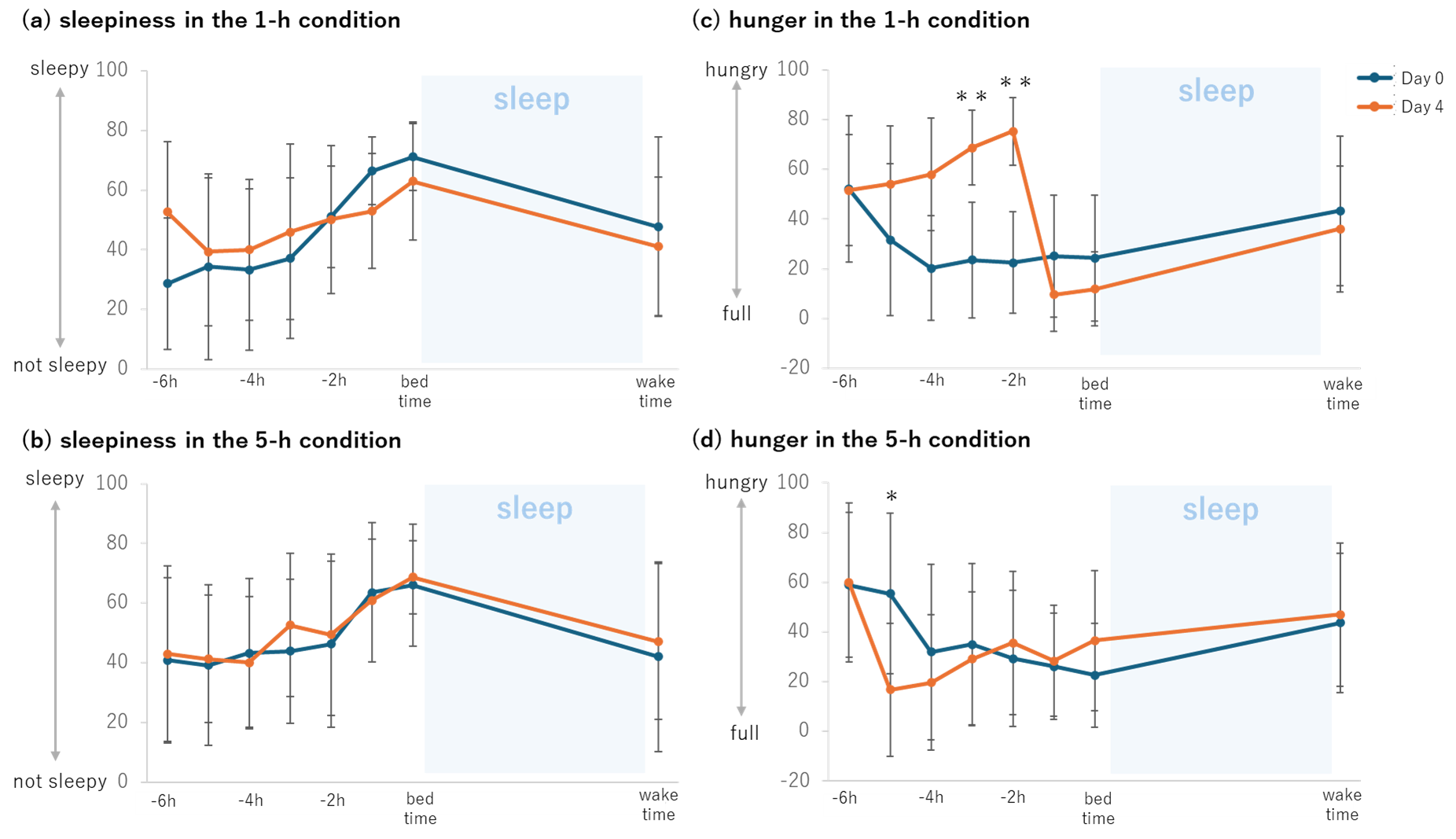
**

**Fig. S1**. Subjective sleepiness and hunger. (a) Sleepiness in the 1-h condition, (b) sleepiness in the 5-h condition, (c) hunger in the 1-h condition, and (d) hunger in the 5-h condition. The blue line represents Day 0, and the orange line represents Day 4. According to the results of a three-way mixed-effects ANOVA, no significant interaction was observed for sleepiness, whereas a significant interaction was observed for hunger. Significance markers were added at points where multiple comparisons revealed significant differences between Day 0 and Day 4. Data are presented as mean ± SD. *p < 0.01, **p < 0.001.
